# Relative Validity and Reproducibility of a Short Food Frequency Questionnaire to Assess Nutrient Intakes of New Zealand Adults

**DOI:** 10.3390/nu12030619

**Published:** 2020-02-27

**Authors:** Cecilia Ho Yan Sam, Paula Skidmore, Sheila Skeaff, Sherly Parackal, Clare Wall, Kathryn E. Bradbury

**Affiliations:** 1 Department of Human Nutrition, University of Otago, P.O. Box 56, Dunedin, New Zealand; samcecilia2010@gmail.com (C.H.Y.S.); sheila.skeaff@otago.ac.nz (S.S.); 2 Department of Medicine, University of Otago, P.O. Box 4345, Christchurch, New Zealand; 3 Department of Social and Preventive Medicine, University of Otago, P.O. Box 56, Dunedin, New Zealand; sherly.parackal@otago.ac.nz; 4 Discipline of Nutrition and Dietetics, University of Auckland, Private Bag 92019, New Zealand; c.wall@auckland.ac.nz; 5 National Institute for Health Innovation, School of Population Health, University of Auckland, Private Bag 92019, New Zealand; k.bradbury@auckland.ac.nz

**Keywords:** food frequency questionnaire, dietary assessment, validity, reproducibility, short food frequency questionnaire, nutrient intake, adults, New Zealand

## Abstract

There is no recent validated short food frequency questionnaire (FFQ) for use in New Zealand (NZ) adults. This study aimed to evaluate the relative validity and reproducibility of a short FFQ in free-living NZ adults aged 30–59 years. A 57-item, semi-quantitative FFQ was developed and pre-tested. During a 12-month study period the FFQ was administrated twice with a 9-month interval between administrations. Four two-day diet records were collected at months 0, 3, 6, and 9 and a blood sample was taken at month 9. Spearman correlations were used to evaluate the validity of the FFQ with the eight-day diet records and selected biomarkers. Cross-classification analysis and the Bland–Altman method were used to assess the agreement between the FFQ and the diet record. Reproducibility over nine months was assessed using intra-class correlations. A total of 132 males and females completed both FFQs, the eight-day diet record, and provided a blood sample. The highest energy-adjusted correlation coefficients were observed for alcohol (0.81), cholesterol (0.61), and carbohydrate (0.61), with the lowest for sodium (0.29), thiamin (0.33), and niacin equivalents (0.34). More than three quarters of the participants were correctly classified into the same or adjacent quartile for most nutrients, with a low proportion of participants being grossly misclassified (<10%). For most nutrients, the limits of agreement from the Bland–Altman analyses were between 50% and 250%. A positive correlation was observed between dietary intakes and plasma biomarkers for all selected nutrients. The FFQ showed moderate to good reproducibility, with almost all reliability coefficients ranging from 0.60 to 0.80. This short FFQ was shown to validly and reliably rank individuals by their habitual intake of most major nutrients, indicating that the FFQ will offer a time-efficient way to assess the nutrient intake of NZ adults in future research.

## 1. Introduction

Sub-optimal diets contribute significantly to the global burden of disease [1]. In New Zealand, diet-related risk factors such as low consumption of fruit and vegetables, wholegrains and omega-3 fatty acids, and high consumption of sodium and trans fat, are estimated to be the leading cause of health loss [2]. This is primarily due to their relationships with cardiovascular disease, but also cancer, and type 2 diabetes and its renal complications [2]. Thus, research into diet as a modifiable risk factor for disease remains a priority. 

The vast majority of nutritional epidemiological studies use food frequency questionnaires (FFQs) as the main measure of dietary assessment because of their ability to prospectively capture habitual dietary intake in an efficient manner. Food frequency questionnaires for use in a new population can be adapted from existing FFQs; however, the performance of each food frequency questionnaire should be evaluated in the intended target population, given inevitable differences in culture, food availability, and food intake between populations [3]. 

Our group recently adapted and validated an FFQ that has been shown to be able to reliably rank New Zealand adults by usual intake of multiple nutrients [4]. This FFQ is a comprehensive tool comprising of 154 items, requiring around 30 to 45 min to complete. The time taken to complete a long FFQ can limit its use in multidisciplinary studies, where typically large volumes of information are collected and the overall participant burden needs to be managed. In addition, a review of FFQ validation studies found that, for most nutrients, FFQs with more items performed similarly or only slightly better at ranking participants than FFQs with fewer items [5,6]. There are no recent short FFQs that have been developed for use in New Zealand adults. The aim of the current study was to develop a short FFQ, with fewer than 100 items, for use in NZ free-living adults (aged 30–59), and to assess its validity and reproducibility for ranking individuals according to their usual intake of multiple nutrients.

## 2. Materials and Methods 

### 2.1. Subjects and Study Design

Ethical approval for the study was obtained from the University of Otago Human Ethics Committee (Ref: 09/078), and all participants gave written and informed consent. We recruited a convenience sample of 151 Dunedin residents, stratified by age and gender, so that there were approximately equal numbers of males and females in each of the following 10-year age groups: 30–39 years, 40–49 years and 50–59 years. Participants were eligible for inclusion in the study if they were healthy (i.e., did not have a chronic disease), non-pregnant, and not on a diet. Participants who were overweight or obese were eligible for the study.

During a 12-month study period, participants were required to attend four clinic visits (at month 0, month 3, month 9, and month 12). At each clinic visit, height and weight were measured by a trained research assistant using a standardised procedure. To assess the reproducibility of the FFQ, participants completed the FFQ on two occasions, nine months apart; half of the participants were randomised to group 1 and completed the FFQ at the month 0 and month 9 clinic visits, and the remaining half were randomized to group 2 and completed the FFQ at month 3 and month 12 visits. Computer generated simple randomization was used. At month 0, all participants completed a questionnaire that collected demographic information. In addition, all participants were given verbal instructions on completing a weighed diet record and were provided with an electronic scale (Salter, Kent, UK) and a diet record package, which consisted of a 2-day diet record (2 dWDR) booklet and a pre-paid envelope for the return of records. Written instructions on weighing and recording were also available in the 2dWDR booklet. Participants were instructed to complete and return the 2dWDR within 2 weeks of the visit; if completed 2dWDRs were not received, participants were followed up by email or telephone. All returned 2dWDRs were checked for completion and a follow-up contact was made when detailed information was required. Participants randomly assigned to group 1 also completed the FFQ at the month 0 clinic visit. At the month 3 clinic visit, the second 2dWDR package was given to all participants. In addition, participants randomly assigned to group 2 completed their first FFQ. The third 2dWDR was sent to all participants by post at month 6. At the month 9 clinic visit, the fourth 2dWDR was given to all participants. Also at the month 9 clinic visit, a total of 20ml fasting blood sample was collected from all participants for biochemical analyses. The participants in group 1 who completed the FFQ at month 0 completed their second FFQ at the month 9 clinic visit, whereas those in group 2 who completed their first FFQ at the month 3 clinic visit repeated it at the month 12 clinic visit. 

### 2.2. Development and Administration of The FFQ

The short FFQ was adapted from the 163-item semi-quantitative FFQ developed by Willett [7,8]. Revisions were made to ensure that all foods listed in the FFQ reflected the local dietary habits according to the 1997 NZ National Nutrition Survey, which was the most recent nationally representative data set available at the time the questionnaire was developed. Two supermarket tours were also undertaken to identify commonly used food names and brands, in order to add this information into the food descriptions.

The major change made to the Willett FFQ was the length of the food list. Foods that are not commonly consumed in NZ were eliminated, and foods with similar nutrient values (e.g., white bread and white tortillas) were grouped into a single item. Where nutritionally similar foods were grouped together into one item, their portion sizes were adjusted to provide similar nutritional composition per portion. For example, a serving size of “potato” was changed from “1 cup” to “1/2 cup” to match the nutrient content of “1/2 cup” “kumara”. Examples of other amendments included naming changes (e.g., “sweet potato” was replaced by “kumara”, the name used in New Zealand). Dietary supplement use was asked in a single yes/no question, and type of fat use was asked in an open-ended question. The modified FFQ was pre-tested in two focus groups of 21 adults aged 30–59 years and subsequent modification was made according to feedback received. The finalised FFQ is designed to assess average consumption of 57 food items over the past 12 months. For the frequency section, the highest two response options (i.e., 4–5 times daily and 6+ daily) employed by the Willett FFQ were merged into one category. Information on quantity is collected using a provided standard portion size and eight frequency options, ranging from “never or less than once per month” to “4–6 times a day”. A list of all 57 food items included in the final FFQ are presented in Table S1.

To calculate nutrients, the New Zealand food composition database (New Zealand FOODfiles, 7th Edition) was used. Each relevant food in a particular food item was weighted according to the data on food consumption patterns from the 2008/09 New Zealand Adult Nutrition Survey. For example, the item “low-calorie drink” was matched to two beverages (diet cola and diet lemonade) in the New Zealand food composition database, and the nutrient composition was weighted as 73% diet cola and 27% diet lemonade as diet cola was 2.7 times more frequently consumed by the 2008/09 New Zealand Adult Nutrition Survey participants than the latter. Nutrient intakes of the participants were calculated by multiplying the frequency of consumption of each food item and the matched nutrient composition for that item.

### 2.3. Weighed Diet Record

A weighted diet record was used as the reference method. Power calculations indicated that eight recording days were necessary to reflect the habitual dietary intake of New Zealand adults and to provide intra-class correlation coefficients (ICC) of 0.95 or higher for all nutrients, including those with high day-to-day variation in intake (e.g., vitamin C and Selenium). Eight days of records (8dWDR) were collected in four blocks of 2 non-consecutive days, approximately three months apart, i.e., one block per season. Equal numbers of participants were randomly assigned to start recording on each of the seven days in a week. Each participant recorded the remaining days following a standard order. Analyses of nutrient intake were performed using the New Zealand FOODfiles based Diet Cruncher software version 1.6.0 (Way Down South Software, Dunedin, New Zealand), which uses the 7th edition of the 2006 New Zealand FOODfiles food composition database. Daily nutrient intake was then computed taking into account the 5:2 ratio of weekdays to weekend days.

### 2.4. Blood Biomarkers

Validation of the dietary intakes of vitamin C, β-carotene, and vitamin E determined from the FFQ and the 8dWDR was made using the respective blood concentrations of ascorbic acid, β-carotene and α-tocopherol. A 20-ml fasting blood sample was collected from each participant at the month 9 clinic visit. Blood samples were allowed to coagulate for 30 min before centrifuging at 1500× *g* for 15 min at 4 °C. Plasma samples were stabilised within 3 hours of collection to minimize degradation of ascorbic acid. All plasma and serum samples were stored at –P 80 °C for up to a year before analysis. A fluorometric assay was undertaken to determine plasma ascorbic acid concentration [9]. The inter-assay coefficient of variation (CV) was 4.7%, and the intra-assay CV at 2, 4, and 5 µg ascorbic acid/ml was 6.7%, 3.2% and 4.0%, respectively. A concurrent liquid chromatographic assay was performed to determine the concentrations of serum β-carotene and α-tocopherol [10]. The inter-assay CVs were 9.1% for β-carotene and 3.5% for α-tocopherol, and the intra-assay CVs were 10.4% for β-carotene and 1.7% for α-tocopherol. All serum samples and standard solutions for β-carotene and α-tocopherol analyses were handled under natural lighting.

### 2.5. Statistical Analyses

All data were analysed using the statistical software Stata 11.0 (Stata Corp, College Station, TX). Intraclass correlation coefficients (ICC) were used to assess the reproducibility between the first and the second administrations of the FFQ (FFQadmin1 vs. FFQadmin2). Spearman correlation coefficients (SCC) were used to assess the relative validity of FFQadmin2 in relation to the 8dWDR and blood biomarkers. Since our FFQ is designed to rank individuals according to their usual nutrient intake, cross-classification of results between FFQadmin2 and the 8dWDR was performed. The percentage of participants grossly misclassified into extreme fourths, and percentage correctly classified into same and adjacent fourths were calculated. The Bland–Altman method [11] was also used to compute the strength of agreement between FFQadmin2 and the 8dWDR for absolute measures of nutrient intakes. To do this, the original FFQ and 8dWDR data was log-transformed to improve normality. Computed mean difference and limits of agreement were back-transformed and presented in percentages. Thus, a percentage mean difference of 100 with 95% confidence intervals including 100 represents prefect agreement. The range of limit of agreement (LoA) indicates an interval that comprises 95% of the differences between the two methods. For all validity analyses, energy adjustment was undertaken according to the residual method described by Willett et al. [12]. An additional adjustment for blood cholesterol was performed to assess the correlation between dietary vitamin E intake and blood α-tocopherol using the residual method.

## 3. Results

Of the 151 enrolled participants, 132 completed an 8dWDR, both administrations of the FFQ and a fasting blood test, providing a completion rate of 87%. No missing values were found in the FFQs from these 132 participants. Of these participants, 51% were female, 81% were of New Zealand European ethnicity, and the mean age was 44 years. 

Table 1 shows mean energy and nutrient intakes derived from the 8dWDR, the two FFQ administrations, and the mean intakes of participants from the 2008/09 New Zealand Adult Nutrition Survey. The mean energy intake from the 8dWDR was 9.2MJ/day, which was similar to 9.0 MJ/day reported by 2008/09 New Zealand Adult Nutrition Survey participants of a similar age using an interview-administered 24 hour recall. In general, the nutrient intakes of participants in the current study were comparable to those in the 2008/09 New Zealand Adult Nutrition Survey. 

Table 2 presents the reproducibility of the FFQ repeat-administrated nine months apart. The lowest ICCs were observed for thiamin (0.53), niacin equivalents (0.55), and zinc (0.56), while the highest ICCs were observed for vitamin C (0.75), alcohol (0.73) and sodium (0.71). The median ICC was 0.65. The validity of the FFQadmin2 in relation to the 8dWDR is also shown in Table 2. The validity coefficients of the energy-adjusted data were higher than the crude data for almost all nutrients. After energy-adjustment, the lowest SCCs were observed for sodium (0.29), thiamin (0.33), and niacin equivalents (0.34), while the highest SCC were found for alcohol (0.81), cholesterol (0.61), and carbohydrate (0.61). The median SCC was 0.49.

Table 3 shows the cross-classification results of the FFQadmin2 and the 8dWDR in ranking participants into fourths according to nutrient intakes. On average, 76.2% participants were correctly classified into the same or adjacent fourths, whereas 5.7% were grossly misclassified into extreme fourths. Energy adjustment improved the mean percentage of correct classification to 80.4% and reduced the mean percentage of gross misclassification to 3.8%. These mean percentages were better than that expected by chance (i.e., 62.5% for correct classification and 12.5% for gross classification) for all nutrients.

Table 4 shows the strength of agreement between FFQadmin2 and the 8dWDR. For most nutrients, the 95% CIs included 100 and thus indicate perfect agreement. After energy adjustment, the mean percentage agreement was similar but the range of LoA was narrowed for all nutrients. For example, the range of LoA for total fat narrowed from 49%–216% to 75%–150% after energy adjustment. Most energy-adjusted estimates of FFQadmin2 fell between half and two times the 8dWDR estimates, indicating a good agreement between the methods.

The mean (SD) concentration of blood biomarkers was 68.7 (22.0) mmol/l for ascorbic acid, 0.59 (0.44) mmol/l for β-carotene, and 26.9 (7.95) mmol/l for α-tocopherol. Table 5 shows the validity of the FFQadmin2 and 8dWDR in relation to blood biomarkers. After energy adjustment, the SCC between the FFQ estimates and the blood biomarkers was 0.29 for ascorbic acid and 0.28 for β-carotene, which was similar to the SCC between the 8dWDR and the blood biomarkers (0.38 for ascorbic acid and 0.30 for β-carotene). Small but positive correlations between each dietary assessment methods and serum alpha-tocopherol were observed. 

## 4. Discussion

In this paper we describe the development of a 57-item FFQ for use in New Zealand adults. We also present the reproducibility and the validation of this short FFQ. The short FFQ showed good relative validity for ranking participants by usual intake of most nutrients. In addition, this short FFQ had good reproducibility with high intra-class correlation coefficients between nutrients from the short FFQ administered twice, nine months apart.

### 4.1. Relative Validity

As is the case for most FFQ, our short FFQ was designed to rank individuals according to their nutrient intakes. When comparing against the 8dWDR, our short FFQ showed acceptable to good validity for all nutrients, given that for most nutrients, the energy-adjusted SCC fell within the commonly reported range of 0.45–0.70 [13,14,15,16,17].

In a review of FFQ validation studies, Molag et al. [5] found that shorter FFQs had significantly lower validity for total fat, vitamin C and protein, but not other nutrients. In a review of Japanese FFQ validation studies Wakai et al. [6], found that longer FFQs tended to have higher validity. These findings suggested that a reduced food list might omit some dietary sources that might be important to distinguish differences in individual dietary intakes. However, after energy adjustment, the SCC of our short FFQ ranged from 0.29 to 0.81, which is similar to the range of 0.24–0.74 for our previously validated 157-item FFQ. This suggests that our short FFQ is highly likely to include the most important dietary sources of nutrients, and is able to capture most information obtained in a considerably longer FFQ.

The use of biomarkers in validating an FFQ should be well-thought out with consideration that concentration biomarkers reflect both intake and metabolism of nutrients rather than simply intake itself [18]. For example, α-tocopherol concentration in blood is closely dependent on the quantity of its lipid-containing carriers [19,20]. Adjustment for total blood cholesterol was undertaken in the current study to eliminate the effects of inter-personal variation in α-tocopherol metabolism [21]. However, we found only a weak SCC between the blood concentrations of alpha-tocopherol and vitamin E intake as assessed by either the short FFQ or the 8dWDR. After energy adjustment, the SCC for plasma ascorbic acid, vitamin C (r = 0.29), serum β-carotene, and β-carotene intake (r = 0.28) were highly comparable to that reported in previous studies [21,22,23,24,25].

In addition to correlation coefficients, we used the Bland–Altman method as a complementary method to assess absolute agreement between an FFQ and its validation references. Apart from alcohol and β-carotene, our FFQ showed a satisfactory agreement for all other nutrients, as shown by the LoA falling between the range of 50%–250%. Previous studies have also found satisfactory LoA for the main macronutrients protein, carbohydrate, and fat, but have similarly reported wide LoA for some micronutrients [26,27]. For example, an Australian version of the Willett FFQ provided wide LoA for alcohol and β-carotene particularly; the FFQ estimates for alcohol ranged from 88% lower to 843% higher and for β-carotene ranged from 27% lower to 790% higher than that measured by a 12-day diet record [26]. These findings indicate the difficulty in obtaining absolute measures using an FFQ, especially for alcohol and β-carotene as observed in the current study. 

### 4.2. Reproducibility

Compared to other FFQs that were repeat-administrated with an approximate 9 month interval, the short FFQ showed a highly comparable, or even greater, reproducibility coefficients for energy and all nutrients [4,8,28,29,30,31]. In addition, all reproducibility coefficients reported in this study fell within the range of 0.47–0.83, which was observed in a 154-item FFQ validated by our team [4]. 

### 4.3. Strengths and Limitations

One of the strengths in this validation study is the use of two referent methods; a weighed diet record and biomarkers. Estimated diet records require portion size estimation, which would introduce errors correlated with the FFQ. The current study adopted an 8dWDR that covered all days in a week and all four seasons in a year, so as to capture as much dietary variation as possible. Thus, the 8dWDR is likely to represent habitual dietary intake and serves as a good referent validation method. 

We used circulating concentrations of β-carotene, ascorbic acid and α-tocopherol as biomarkers of intake of β-carotene, vitamin C and vitamin E, respectively. Although these biomarkers are affected by metabolism, personal characteristics and lifestyle factors [18], observational studies [32,33], and controlled feeding studies [34] have shown that circulating concentrations of these vitamins are useful biomarkers of intake.

Nevertheless, this is a relative validation study and the referent methods we used are not without bias. When completing a weighed diet record, participants may change their typical diet or not record all food that they ate [35]. In addition, we used a single blood sample for biomarker measurements and it has been shown that there can be significant within-person variation in biomarkers [36].

To assess reproducibility, our FFQ was administered nine months apart. The adopted interval was pragmatically chosen to allow for an appropriate length of time to dilute learning effects and to minimize variance from true dietary changes. 

Compared to FFQ collected in the same season one year apart, our FFQ repeat-administered in different seasons might yield lower reproducibility coefficients, as seasonal reporting bias has been documented in FFQs [37]. However, our FFQ showed a highly comparable reproducibility coefficient for energy to FFQ that repeat-administrated at one year, indicating the ability of our FFQ to capture habitual dietary intake over a year. 

Finally, one of the main challenges of FFQ validation studies is to recruit a nationally representative sample of the target population. According to the latest NZ census report, Māori and Pacific Islanders comprised 22% of the NZ population, which is markedly higher than the 4% recruited in the current study. Since Māori and Pacific Islanders are likely to have different dietary patterns and higher BMI than their European counterpart, further evaluation of the use of the FFQ for Māori or Pacific Island populations is desirable.

## 5. Conclusions

The results of this validation and reproducibility study indicate the ability of our short FFQ to discriminate between New Zealand adults with low and high nutrient intakes, which is the main focus in most epidemiological studies. Because it contains just 57-items, this short FFQ is suitable for use in a setting that has limited time and resources available to collect dietary information.

## Figures and Tables

**Table 1:** Mean (SD) daily intake for energy and selected nutrients among participants in this study ^1^ and the 2008/09 Adult Nutrition Survey (ANS).

	Short FFQadmin ^1^		ShortFFQadmin ^1^		8dWDR		ANS
Nutrient	Mean	SD	Mean	SD	Mean	SD	Mean
Energy (MJ)	9.7	2.8	9.4	2.8	9.2	2.2	9.1
Protein (g)	99.3	29.6	95.1	31.7	90.1	23.6	88
Total fat (g)	82.6	26.3	81.0	27.5	78.1	25.7	83
Saturated fat (g)	32.5	11.7	31.6	12.1	29.6	12.2	32.4
Monounsaturated fat (g)	29.4	9.4	29.1	9.9	27.4	9.6	30.5
Polyunsaturated fat (g)	12.9	5.1	13.0	5.4	13.0	6.0	11.7
Cholesterol (mg)	261.3	113.4	256.7	124.5	267.1	139.2	281
Carboyhdrate (g)	277.1	100.2	267.1	93.9	268	75.7	250
Sucrose (g)	48.3	23.2	45.2	20.0	46.0	19.5	54.2
Fructose (g)	28.3	13.6	27.4	12.9	26.0	11.0	21.4
Fibre (g)	30.2	11.1	30.1	11.6	27.7	8.8	20.3
Alcohol (g)	10.0	15.2	10.5	15.7	11.0	17.5	14.0
Total vitamin A (µg)	1079	458	1085	541	1064	776	879
β-carotene (µg)	4801	2586	4840	3089	4087	2580	2838
Thiamin (mg)	1.7	1.6	1.6	1.2	1.9	1.0	1.5
Riboflavin (mg)	2.5	1.0	2.3	0.9	2.1	0.7	2.0
Niacin equivalents (mg)	41.3	11.1	39.8	12.2	37.9	10.6	36.9
Vitamin B6 (mg)	2.2	0.8	2.1	0.8	2.1	0.0	2.1
Folate (µg)	551	255	570	308	476	191	-
Vitamin B12 (µg)	4.8	2.4	4.3	2.1	4.5	2.9	4.4
Vitamin C (mg)	124.2	60.5	119.3	63.6	117.9	54.1	108
Vitamin E (mg)	13.0	3.8	12.7	4.1	10.9	4.5	10.6
Calcium (mg)	1171	555	1095	520	962	309	871
Potassium (mg)	1772	585	1682	584	3781	990	3161
Iron (mg)	14.1	4.9	13.8	4.6	16.8	7.3	11.9
Selenium (µg)	61.4	30.3	57.5	23.4	56.9	27.5	59.5
Sodium (mg)	2574	912	2536	936	2761	898	-
Magnesium (mg)	423	122	408	122	402	129	-
Zinc (mg)	13.0	3.8	12.4	3.9	11.7	3.2	11.3

8dWDR = 8-day weighed diet record; shortFFQadmin1 = 1st administration of the short FFQ used in this study; shortFFQadmin2 = 2nd administration of the short FFQ used in this study; ANS = 2008/09 New Zealand Adult Nutrition Survey.^1^ Intake from supplements was not included.

**Table 2:** Intraclass correlation coefficients (ICC)^1^ and Spearman correlation coefficients (SCC)^2^ of the short FFQ for crude and energy-adjusted nutrient intakes.

Nutrient	Reproducibility ^1^	Relative Validity ^2^	
		Crude	Energy-Adjusted
Energy	0.63	0.18	-
Protein	0.59	0.29	0.54
Total fat	0.63	0.30	0.54
Saturated fat	0.63	0.34	0.54
Monounsaturated fat	0.60	0.25	0.39
Polyunsaturated fat	0.68	0.34	0.37
Cholesterol	0.65	0.60	0.61
Carboydrate	0.66	0.37	0.61
Sucrose	0.57	0.45	0.48
Fructose	0.68	0.52	0.58
Fibre	0.70	0.38	0.56
Alcohol	0.73	0.85	0.81
Total vitamin A	0.67	0.37	0.42
β-carotene	0.67	0.37	0.43
Thiamin	0.53	0.32	0.33
Riboflavin	0.66	0.44	0.52
Niacin equivalents	0.55	0.23	0.34
Vitamin B6	0.65	0.32	0.51
Folate	0.70	0.46	0.48
Vitamin B12	0.66	0.50	0.50
Vitamin C	0.75	0.38	0.47
Vitamin E	0.67	0.36	0.39
Calcium	0.66	0.51	0.58
Potassium	0.65	0.31	0.56
Iron	0.59	0.26	0.35
Selenium	0.59	0.36	0.39
Sodium	0.71	0.29	0.29
Magnesium	0.65	0.35	0.54
Zinc	0.56	0.20	0.43

^1^ ICC between the first and second administrations of the short FFQ. ^2^ SCC between the second administration of the short FFQ and the 8dWDR.

**Table 3:** Cross-classification of subjects by quartiles of nutrient intakes between the Food Frequency Questionnaire (FFQ) and the 8-day weighted diet record (8dWDR).

	Crude		Energy-Adjusted	
Nutrient	Correct Classification (%) ^1^	Gross Misclassification (%) ^2^	Correct Classification (%) ^1^	Gross Misclassification (%) ^2^
Energy	68.2	7.4	-	-
Protein	68.2	5.9	81.5	3.0
Total fat	72.6	7.4	85.9	3.0
Saturated fat	76.3	6.7	88.9	3.7
Monounsaturated fat	72.6	8.9	73.4	4.4
Polyunsaturated fat	74.8	8.2	74.8	5.9
Cholesterol	85.9	2.2	83.7	2.2
Carbohydrate	78.5	5.9	85.2	1.5
Sucrose	77.1	4.4	80.0	4.4
Fructose	82.2	3.0	81.5	1.5
Fibre	79.3	5.2	85.2	3.0
Alcohol	98.5	0.0	95.7	0.0
Total vitamin A	76.3	7.4	77.1	5.2
β-carotene	73.4	6.7	78.5	5.2
Thiamin	77.1	7.4	76.3	6.7
Riboflavin	77.8	3.0	83.7	4.4
Niacin equivalents	68.2	8.9	68.9	2.2
Vitamin B6	74.1	6.7	82.2	3.7
Folate	77.1	3.7	81.5	3.0
Vitamin B12	81.5	4.4	85.2	5.9
Vitamin C	76.3	4.4	80.0	3.0
Vitamin E	77.1	7.4	75.6	6.7
Calcium	80.8	0.7	83.0	3.7
Potassium	74.1	5.2	81.5	1.5
Iron	70.4	7.4	76.3	3.7
Selenium	77.0	5.9	77.1	5.9
Sodium	76.3	9.6	72.6	6.7
Magnesium	72.6	4.4	78.5	2.2
Zinc	66.7	8.1	77.8	4.4

^1^ Percentage of participants classified into the same or adjacent fourth of nutrient intake (intake from supplements not included).^2^ Percentage of participants classified into the extreme fourth of nutrient intake (intake from supplements not included).

**Table 4:** Strength of agreement using Bland–Altman method between nutrient intake derived from the Food Frequency Questionnaire (FFQ) and the 8-day weighted diet record (8dWDR).

	Crude			Energy-Adjusted		
Nutrient	Mean (%) ^1^	(95% CI)	Limits of Agreement (%) ^2^	Mean (%) ^1^	(95% CI)	Limits of Agreement (%) ^2^
Energy	100	95, 106	54–186	-	-	-
Protein	104	99, 110	57–190	107	104, 110	79–144
Total fat	103	97, 110	49–216	106	103, 109	75–150
Saturated fat	107	100, 115	47–245	111	106, 115	69–177
Monounsaturated fat	106	99, 114	47–238	109	105, 114	68–176
Polyunsaturated fat	100	93, 108	42–237	103	98, 108	57–187
Cholesterol	99	92, 106	44–221	101	95, 108	51–203
Carbohydrate	97	91, 103	49–194	100	98, 102	77–130
Sucrose	96	89, 105	38–247	100	94, 106	49–202
Fructose	103	96, 111	43–248	106	100, 112	54–209
Fibre	106	99, 113	50–226	109	105, 114	68–175
Alcohol	116	96, 140	13–1021	118	98, 142	14–984
Total vitamin A	102	94, 112	38–276	105	97, 114	43–256
β-carotene	119	107, 134	32–450	122	110, 136	36–417
Thiamin	83	76, 92	28–245	86	79, 93	34–218
Riboflavin	104	98, 111	52–209	106	101, 112	61–186
Niacin equivalents	105	100, 111	56–196	108	104, 111	72–160
Vitamin B6	98	91, 106	42–227	101	96, 107	55–187
Folate	114	105, 123	47–277	117	110, 125	58–236
Vitamin B12	100	92, 109	37–270	102	95, 111	41–259
Vitamin C	97	88, 106	33–280	99	92, 108	39–253
Vitamin E	117	110, 125	58–237	120	114, 126	70–206
Calcium	108	101, 115	49–236	111	105, 117	61–202
Potassium	101	96, 107	53–191	104	101, 107	75–143
Iron	83	78, 90	36–193	85	81, 90	48–152
Selenium	103	95, 112	42–256	106	99, 113	47–237
Sodium	90	84, 97	40–206	93	89, 98	54–160
Magnesium	101	95, 107	52–194	103	100, 107	69–155
Zinc	104	99, 111	53–205	107	104, 111	74–154

^1^ Mean % agreement= (nutrient intakes of FFQ/nutrient intake of 8dWDR) × 100%. ^2^ The width of the Limits of Agreement represents the range in which 95% of the differences between the FFQ and the 8dWDR are expected to fall.

**Table 5:** Spearman correlation coefficients (SCC)^1^ for the relative validity of short food frequency questionnaire (FFQ)^2^ compared to blood biomarkers for B-carotene, and vitamins C and E.

	Short FFQ		8-Day Weighted Diet Record	
Nutrient	Crude	Adjusted	Crude	Adjusted
β-carotene	0.30	0.28	0.30	0.30
Vitamin C	0.20	0.29	0.36	0.38
Vitamin E	−0.02	0.04	−0.08	0.09

^1^ SCC for total nutrient intake including relevant supplement data. ^2^ The biomarker of β-carotene (µg/day) intake is β-carotene (µmol/L); vitamin C (mg/day) intake is plasma ascorbic acid (µmol/L); and vitamin E (mg/day) intake is serum α-tocopherol (µmol/L).

## Supplementary Materials

The following are available online at https://www.mdpi.com/2072-6643/12/3/619/s1, Table S1: List of food items included in the Short Food Frequency Questionnaire.
