# Supplementary material for: Relative Validity and Reproducibility of a Short Food Frequency Questionnaire to Assess Nutrient Intakes of New Zealand Adults
Source: Nutrients. 2020 Feb 27;12(3):619. doi: 10.3390/nu12030619 (PMC7146506; doi:10.3390/nu12030619)
Supplement: Supplementary file 1 [file nutrients-12-00619-s001.pdf]

Sam et al. Relative validity and Reproducibility of a Short Food Frequency Questionnaire to Assess Nutrient Intake of New Zealand Adults.

Table S1. List of food items included in the Short Food Frequency Questionnaire.

**Food group**	**Food item(s)**
Dairy foods	
	Milk (include milk in hot drinks, cereals, creamed soups etc) e.g. Cow, Soy*
	Ice cream
	Yoghurt
	Low fat cheese e.g. Cottage, Ricotta, Low Fat Cheddar
	Cheese e.g. Cheddar, Edam, Tasty, Mozzarella, Brie, Camembert
	Cream based dairy e.g. Cream, Sour cream, Cream cheese
Fruits	
	Bananas
	Apples/Pears
	Citrus fruit: Oranges, Mandarins, Grapefruit, Lemons
	Stone fruit e.g. Apricots, Plums, Nectarines, Peaches
	Berries fresh, frozen or canned e.g. Strawberries, Blueberries
	Dried fruit e.g. Raisins, Sultanas, Prunes
	Other fruit e.g. Kiwifruit, Grapes, Feijoa, Pineapple, Mango, Rhubarb, Tamarillos, Guava, Pawpaw, Melon
Vegetables	
	Tomatoes (fresh, canned), tomato based sauce
	Beans or legumes e.g. Green beans, Runner beans, Baked beans, Lentils, Chickpeas
	Salad Greens e.g. Lettuce, Cucumber, Celery, Rocket
	Other Greens: Broccoli, Cauliflower, Spinach, Silverbeet, Cabbage, Brussel Sprouts, Bok choy, Chinese cabbage, Watercress, Puha
	Onion, Leeks
	Potatoes, Kumara, Pumpkin
	Other Root Vegetables e.g. Carrot, Beetroot, Parsnip, Turnips, Yams
	Other Vegetables e.g. Corn, Mushrooms, Asparagus, Courgette, Eggplant, Capsicum, Peas, Coleslaw
Eggs, Meat, Etc	
	Eggs
	Sausages, Hotdogs
	Salami, Ham, Luncheon, Bacon or other processed meat
	Beef, Pork or Lamb e.g. mince, roast, steak, stew, casserole, lasagne, frozen dinners, etc
	Meat pie, 2 sausage rolls
	Chicken and other poultry
	Tuna, Salmon, Sardines, Mackerel
	Fried fish, Battered fish, Breaded fish, Fish fingers

	Other Fish and Seafood e.g. Cod, Sole, Hoki, Gurnard, Shrimp, Mussels, Oysters, Crayfish
Breads, cereals, starches	
	High Fibre Cereals e.g. Porridge, Muesli, Bran Flakes, All Bran
	Other Cold Breakfast Cereal e.g. Light ‘n’ Tasty, Special K, Weetbix
	White Bread including Sliced, Tortillas, Pita etc
	Wholemeal or multigrain Bread including Sliced, Tortillas, Pita etc
	Crackers, Crispbread e.g. Vitawheat, Cruskits
	Pancakes, Waffles, Sweet Buns, Scones
	Brown Rice/Wholemeal Pasta
	White rice / Couscous
	Other Pasta e.g. Spaghetti, Spirals, Instant noodles, Tinned
Fastfoods	
	Hot chips/French fries
	Potato Chips/Crisps or Corn chips
	Pizza/Hamburgers
	International Takeaway e.g. Chinese, Thai, Turkish, Indian, Japanese etc
Beverages	
	Low-calorie drink, e.g. Diet Coke, Coke Zero, Sprite Zero
	Sweet drinks e.g. Sprite, Coke, Fruit juice, Raro, Cordial
	Alcoholic Beverages e.g. Beer, Wine, Spirits
	Water: Bottled, Sparkling, or Tap
	Tea/Coffee
Sweets, baked goods, miscellaneous	
	Chocolate/Chocolate bars
	Sweets, Lollies
	Biscuits
	Cake, Brownie, Slice, Croissant, Pie, Danish, Brioche, Milk Pudding, Muesli bars
	Jam, Preserves, Syrup, Honey
	Nuts (include Peanut butter), seeds
	Oils e.g. Vegetable oil, Olive oil, Mayonnaise, Salad dressing, include frying
	Fats e.g. Butter or Margarine, used as spreads or in cooking, excluding baking
	Iodized salt, at table and in cooking

*Participants were asked in a separate question what type of milk they usually used.
